# Genomic insights into somatic mutations from occupational exposure to low-dose ionizing radiation

**DOI:** 10.5271/sjweh.4317

**Published:** 2026-11-01

**Authors:** Kihun Kim, Junho Kang, Dong Min Lim, Seongjae Jang, Younghyun Lee, Yang Hee Lee, Hyo Jin Yoon, SuSan Yang, Seung Hyun Kim, Dalnim Lee, Won Il Jang, Hyoju Sung, Min-Jung Kim, Eunjeong Son, Hyung-Sik Kim, Dongjun Lee, Hae Ryoun Park, Songwon Seo, Yun Hak Kim

**Affiliations:** 1 Department of Occupational & Environmental Medicine, Pusan National University Yangsan Hospital, Yangsan, Republic of Korea.; 2 Research Institute for Convergence of Biomedical Science & Technology, Pusan National University Yangsan Hospital, Yangsan, Republic of Korea.; 3 Department of Research, Keimyung University Dongsan Medical Center, Daegu, Republic of Korea.; 4 Medical Research Institute, Pusan National University, Yangsan, Republic of Korea.; 5 National Radiation Emergency Medical Center, Korea Institute of Radiological & Medical Sciences, Seoul, Republic of Korea.; 6 Department of Medical Sciences, Graduate School, Soonchunhyang University, Asan, Chungnam, Republic of Korea.; 7 Department of Biomedical Laboratory Science, College of Medical Sciences, Soonchunhyang University, Asan, Chungnam, Republic of Korea.; 8 Institute for Molecular Metabolism Innovation, Soonchunhyang University, Asan 31538, Republic of Korea; 9 FNCT Biotech, Inc. Seoul, Republic of Korea.; 10 Division of Respiratory & Allergy, Department of Internal Medicine, Pusan National University Yangsan Hospital, Yangsan, Republic of Korea.; 11 Department of Oral Biochemistry, Dental & Life Science Institute, School of Dentistry, Pusan National University, Yangsan, Republic of Korea.; 12 Department of Convergence Medicine, School of Medicine, Pusan National University, Yangsan, Republic of Korea.; 13 Transplantation Research Center, Research Institute for Convergence of Biomedical Science & Technology, Pusan National University Yangsan Hospital, Yangsan, Republic of Korea.; 14 Department of Periodontology & Dental Research Institute, Pusan National University Dental Hospital, Yangsan, Republic of Korea.; 15 Periodontal Disease Signaling Network Research Center, School of Dentistry, Pusan National University, Yangsan, Republic of Korea.; 16 Department of Oral Pathology, Dental & Life Science Institute, Pusan National University, Yangsan, Republic of Korea; 17 Department of Biomedical Informatics School of Medicine, Pusan National University, Yangsan, Republic of Korea.; 18 Department of Anatomy, School of Medicine, Pusan National University, Yangsan, Republic of Korea.

**Keywords:** dose–response relationship, radiation worker, whole genome sequencing

## Abstract

**Objectives:**

Low-dose ionizing radiation (LDIR, ≤100 mGy) is a public health concern due to its extensive use in diagnostic and therapeutic imaging. This study examined somatic variants (SV) among Korean industrial radiographers exposed to LDIR using whole-genome sequencing (WGS).

**Methods:**

WGS data from 65 workers (mean age 36.7 years) collected between 2016 and 2023 were analyzed. Participants had a mean employment duration of 12.7 years and an average cumulative radiation dose of 33.9 mSv from National Dose Registry records. SV were identified via the GATK Mutect2 single-sample workflow applied to peripheral blood-derived DNA and sequentially filtered using standard pipelines, including FilterMutectCalls, population frequency, recurrent artifacts, Funcotator annotation, and manual Integrative Genomics Viewer review.

**Results:**

A total of 105 170 somatic variants were identified, with a median of 1744 variants per individual, approximately 98% of which were single nucleotide variants. Cumulative radiation dose showed a significant positive correlation with total SV burden (R=0.32, P=0.013), including both coding (R=0.34, P=0.008) and non-coding regions (R=0.31, P=0.015). Age, smoking, alcohol consumption, hypertension, and hyperlipidemia were not significantly associated with variant burden.

**Conclusions:**

These WGS-based findings provide preliminary insight into blood-derived SV patterns among occupationally exposed radiation workers. Given the small sample size, blood-only design, and absence of matched unexposed controls, the findings should be interpreted as hypothesis-generating and require validation in larger longitudinal studies with comprehensive exposure assessment.

Ionizing radiation comprises particles and photons possessing sufficient energy to ionize atoms within the human body, thereby triggering chemical alterations that could hold biological significance for cellular function (1). Low-dose ionizing radiation (LDIR), which has become an increasing concern in public health, especially in light of the recent significant growth in diagnostic and therapeutic imaging, is typically characterized by a radiation dose of 100 mSv or less (≤100mGy) (2). The quantitative assessment of individual radiation exposure has historically been centered on the health effects resulting from moderate to high-dose acute exposures experienced by survivors of the atomic bombings in Japan. However, there is an ongoing effort to extrapolate and quantify the excess relative risk for health hazards associated with low-dose or protracted exposures, occurring in occupational and general settings (2).

Cancer remains the most extensively studied health outcome associated with LDIR. Potential heritable genetic effects of ionizing radiation have also been investigated, mainly in experimental and animal germline studies, but direct evidence in occupationally exposed human populations remains limited (3–5). These germline findings should be distinguished from the blood-derived somatic variants evaluated in the present study. In addition to cancer, low-dose and low-dose-rate ionizing radiation has been investigated in relation to several non-cancer outcomes, including cardiovascular disease, neurological outcomes, immune and inflammatory alterations, and cataracts, although the strength of evidence and dose-response relationships remain uncertain across endpoints (6).

The emergence of high-throughput sequencing technologies, particularly whole genome sequencing (WGS), has enabled base-resolution characterization of somatic mutagenesis in irradiated cells and organisms. WGS studies in murine models have revealed that ionizing radiation induces increases in point mutations and structural variants, with dose-dependent effects demonstrated in several studies (4, 7), and consistent mutagenic responses observed even at a single low dose (5). These studies provide critical insight into tissue-specific mutational patterns and underlying repair defects. Human WGS studies of radiation-associated mutagenesis have largely focused on cancer patients exposed to radiotherapy or radiation-associated malignancies (8, 9). In contrast, evidence from exposed individuals without overt cancer remains limited, and previous studies in such populations have mainly examined germline or de novo mutations and cytogenetic alterations rather than blood-derived somatic variant burden (10–13).

Radiation workers may be exposed to ionizing radiation through the use of radiation-emitting technologies, including computed tomography, fluoroscopy, interventional radiology systems, and nuclear medicine imaging devices, increasing interest in the long-term health implications of occupational low-dose exposure (14). Recent epidemiological studies have yielded inconsistent results. A multinational cohort study of 309 932 nuclear industry workers from France, the United Kingdom, and the United States reported a positive association between cumulative external radiation dose and solid cancer mortality, with an estimated excess relative rate per Gy that was higher than risk estimates currently used in some radiation protection frameworks (15). The authors also reported no evidence that the dose-response estimate was lower among workers with cumulative doses below 100 mGy. However, in South Korea, no significant association was found between occupational radiation dose and the incidence of solid cancer or leukemia among 93 920 diagnostic radiation workers (16). Another cohort study involving 272 radiation medicine staff revealed an increased lifetime attributable risk of thyroid cancer and leukemia associated with occupational radiation exposure, although the overall assessed risk remained low (17).

Whole genome sequencing (WGS) is a powerful tool for detecting somatic mutations at base-pair resolution (8). Previous studies have used WGS to identify mutational signatures in cancer patients who received radiotherapy, and in irradiated cell lines or animal models (8, 9, 18). However, WGS-based studies investigating low-dose radiation effects in healthy human populations remain scarce. Therefore, the aim of this study was to investigate mutational burden in Korean industrial radiographers through WGS.

## Methods

### Study participants and data collection

Data used in this study were derived from the previous study of Korean industrial radiographers, and the enrollment process of the study population, blood collection, and chromosome aberration analysis are described in detail elsewhere (13). In brief, the study population of the previous study was composed of 120 industrial radiographers, and a self-administered survey including demographics and work history was conducted with blood collection for the chromosome aberration analysis. Occupational radiation doses (ie, effective dose) of the study population were obtained from the National Dose Registry. Of the 120 industrial radiographers enrolled in the study, data from 65 individuals who provided written informed consent for the secondary use of their personal data and biological specimens were included in the present analysis. Peripheral blood samples were collected from these participants between March 5 and June 27, 2018, as part of a structured research protocol with ethical approval (13). Genomic DNA was extracted from the collected samples and subjected to WGS. To reduce potential confounding at the cohort-design stage, individuals with known malignancy, hematologic disease, or major systemic conditions that could substantially influence somatic mutation burden were excluded.

### WGS library construction and sequencing

Sequencing libraries were prepared from 100 ng genomic DNA using the TruSeq Nano DNA High Throughput Library Prep Kit (Illumina) according to the manufacturer’s protocol. Paired-end sequencing (2×150 bp) was performed on an Illumina NovaSeq platform.

### Data preprocessing of the WGS data

Read quality was assessed using FastQC (v0.11.9), and adapter trimming was performed using Cutadapt (v1.15). Reads were aligned to the GRCh38 reference genome using BWA-MEM (v0.7.15), and alignment files were processed with samtools (v1.7). Duplicate reads were marked using Genome Analysis Toolkit (GATK) MarkDuplicatesSpark (v4.2.4.1). Base quality score recalibration was performed using GATK BaseRecalibrator/ApplyBQSR with known variant sites from dbSNP_146, HapMap 3.3, and the 1000 Genomes resources.

### Somatic variant calling

Somatic variant calling was performed using GATK (v4.2.4.1) Mutect2 (19) in single-sample mode without a matched control sample. A panel of normals derived from the 1000 Genomes Project and the gnomAD germline resource were used to reduce likely germline variants and recurrent technical artifacts. Variant filtering was performed using FilterMutectCalls, and variants were annotated using Funcotator with GATK hg38 resources. High-confidence somatic variants were retained after excluding likely germline variants and technical artifacts with the following characteristics (1): variant allele frequency of 1% or higher in the panel of normals (2), three or more mismatched bases in variant-supporting reads (3), overlap with common artifact or germline variant sites (4), recurrent presence as error reads in other samples (5), depth of coverage less than 10 in the blood-derived DNA sample, and (6) average base quality score below 20. The remaining candidate variants were subsequently reviewed manually using Integrative Genomics Viewer (IGV) (20, 21).

### Analysis of somatic variants

Somatic variants were analyzed using a previously validated pipeline (22–25). Variants were summarized by genomic context (coding vs non-coding), functional consequence, and variant class (eg, SNV and indels), and per-sample variant burdens were calculated. Associations between variant burden and cumulative dose or age were assessed using Pearson correlation, and group comparisons (smoking, alcohol use, hypertension, hyperlipidemia) were performed using two-sided Wilcoxon rank-sum tests. Multivariable linear regression was used to estimate the association between cumulative radiation dose and mutation burden after adjustment for covariates (smoking, age, alcohol consumption, hypertension, hyperlipidemia). Available covariates were selected based on their potential relevance to somatic mutation burden or hematopoietic health, including age, smoking, alcohol consumption, hypertension, and hyperlipidemia. However, detailed information on non-occupational ionizing radiation exposure, including diagnostic imaging and therapeutic radiation procedures, as well as comprehensive medication histories and other underlying health conditions, was not available in the present dataset. Hypertension and hyperlipidemia were included as exploratory cardiometabolic covariates because these conditions may reflect systemic inflammatory or oxidative-stress states that could influence hematopoietic dynamics and somatic mutation accumulation. These variables were not considered primary exposures but were included to reduce potential confounding using the available clinical information. Statistical analyses were performed in R (v4.0.2), with two-sided P-values <0.05 considered statistically significant. Mutational signature analysis was conducted using maftools and BSgenome.Hsapiens.NCBI.GRCh38: trinucleotide matrices were constructed, de novo signatures were extracted using non-negative matrix factorization (NMF), and signatures were matched to COSMIC v3.3 using cosine similarity; the number of signatures was selected based on cophenetic correlation. The biological relevance of the identified variants was interpreted based on computational variant annotation and descriptive mutational signature matching. No experimental functional validation, pathway-level functional assay, or mechanistic validation of the identified variants or mutational signatures was performed.

## Results

### Characteristics of the study participants

Demographic and occupational characteristics of the study participants are summarized in table 1, and sample-level participant characteristics and exposure variables are provided in Dataset S1. All participants were men, with a mean age of 36.7 years (SD=6.3; range 20–51 years), and more than half were ever-smokers. The mean duration of employment was 12.7 years (SD=7.0), and the mean cumulative occupational radiation dose was 33.9 mSv (SD=29.6). Detailed dose metrics derived from physical and biological dosimetry are presented in supplementary table S1. Badge-recorded external doses showed a right-skewed distribution and increased with longer aggregation windows. Cytogenetic biodosimetry of peripheral lymphocytes revealed low but measurable absorbed-dose estimates, with substantial inter-individual variability, indicating heterogeneous radiation exposure across workers and time periods.

**Table 1:** Characteristics of the study participants (N=65)^a^. [SD=standard deviation]

Characteristics	N (%)	Mean (SD)	Range
Birth year			
≤1969	5 (7.7)		
1970–1979	12 (18.5)		
1980–1989	45 (69.2)		
≥1990	3 (4.6)		
Smoking status			
Smoker	44 (67.7)		
Non-smoker	21 (32.3)		
Alcohol consumption			
No	8 (12.3)		
Yes	57 (87.7)		
Hypertension status			
Hypertension	6 (9.2)		
Non-hypertension	59 (90.8)		
Hyperlipidemia status			
Hyperlipidemia	6 (9.2)		
Non-hyperlipidemia	59 (90.8)		
Age at blood collection (years)		36.69 (6.28)	20–51
Duration of employment (years)		12.69 (6.98)	0.5–32.33
Cumulative effective dose (mSv)		33.94 (29.62)	1.13–133.72

^a^ Participant characteristics were partially derived from a previous study (13) with additional variables newly analyzed in the present study.

### Distribution and classification of somatic variants in Korean radiation workers

To evaluate the accumulation of somatic variants (SV) associated with low-dose occupational radiation exposure, whole-genome sequencing was performed on 65 Korean industrial radiographers. After initial variant calling, a multi-step filtering strategy was applied to remove low-quality calls, common germline variants, and recurrent artifacts, yielding a final set of 105 170 high-confidence somatic variants, corresponding to 0.578% of the initial variant candidates (figure 1). The complete list of high-confidence somatic variants is provided in Dataset S2.

**Figure 1:**
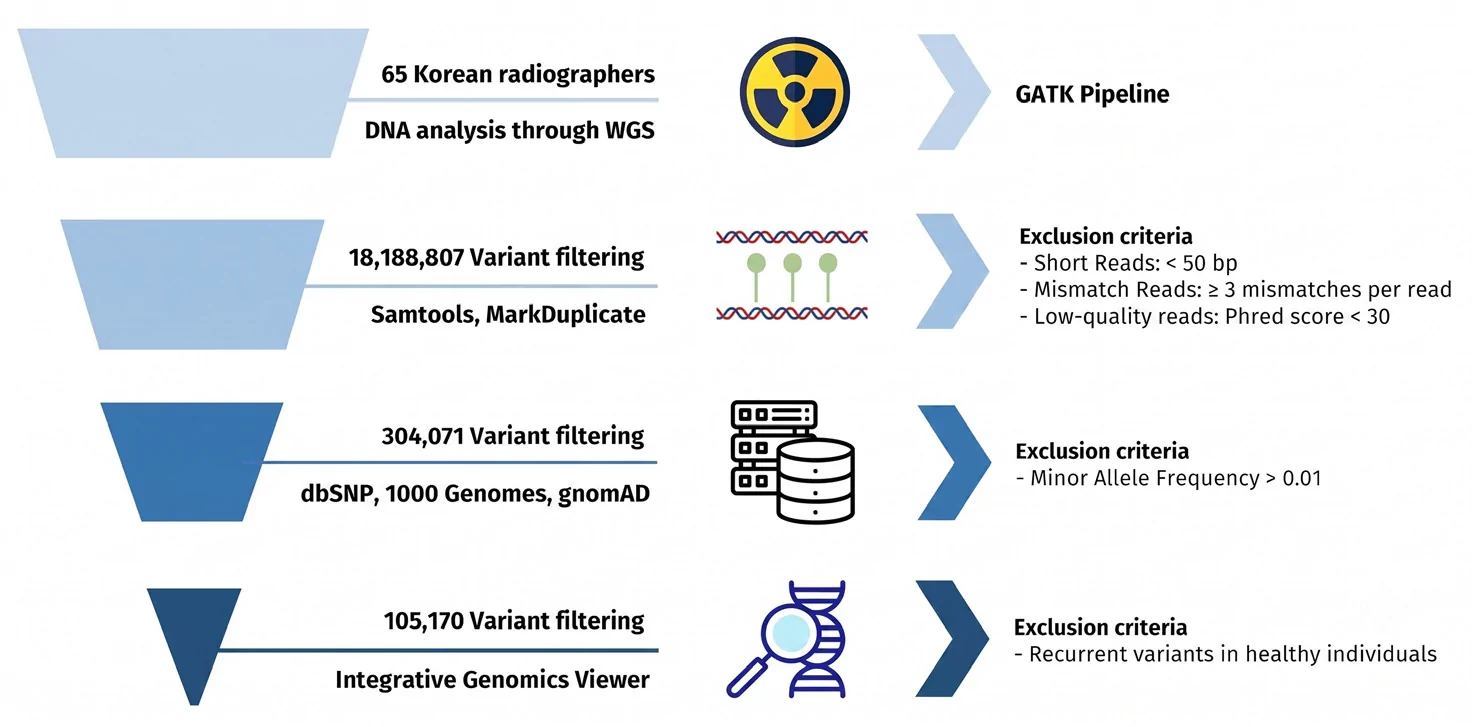
Workflow of somatic variant filtering, outlining the steps involved in filtering somatic variants using the GATK pipeline. The initial step involves the inclusion of high-quality reads with a Phred score >30, excluding short (<50 bp) and mismatch (≥3 mismatches per read) reads. This resulted in 18 188 807 candidate variants. Subsequent variant filtering using databases such as dbSNP, 1000 Genomes, and gnomAD reduced the number to 304 071 variants by removing variants with a MAF >0.01. The final filtering step excludes recurrent variants in healthy individuals, resulting in 105 170 somatic variants included in the analysis.

SV were detected across both coding and non-coding regions of the genome. In coding regions, the median number of SV per individual was 241 (range: 4–276), with missense mutations being the most frequent functional class (figure 2A,B). Single nucleotide variants (SNV) constituted the predominant variant type, followed by small insertions and deletions (figure 2E). The most common nucleotide substitutions among coding SNV were C>T and G>A (figure 2F).

**Figure 2:**
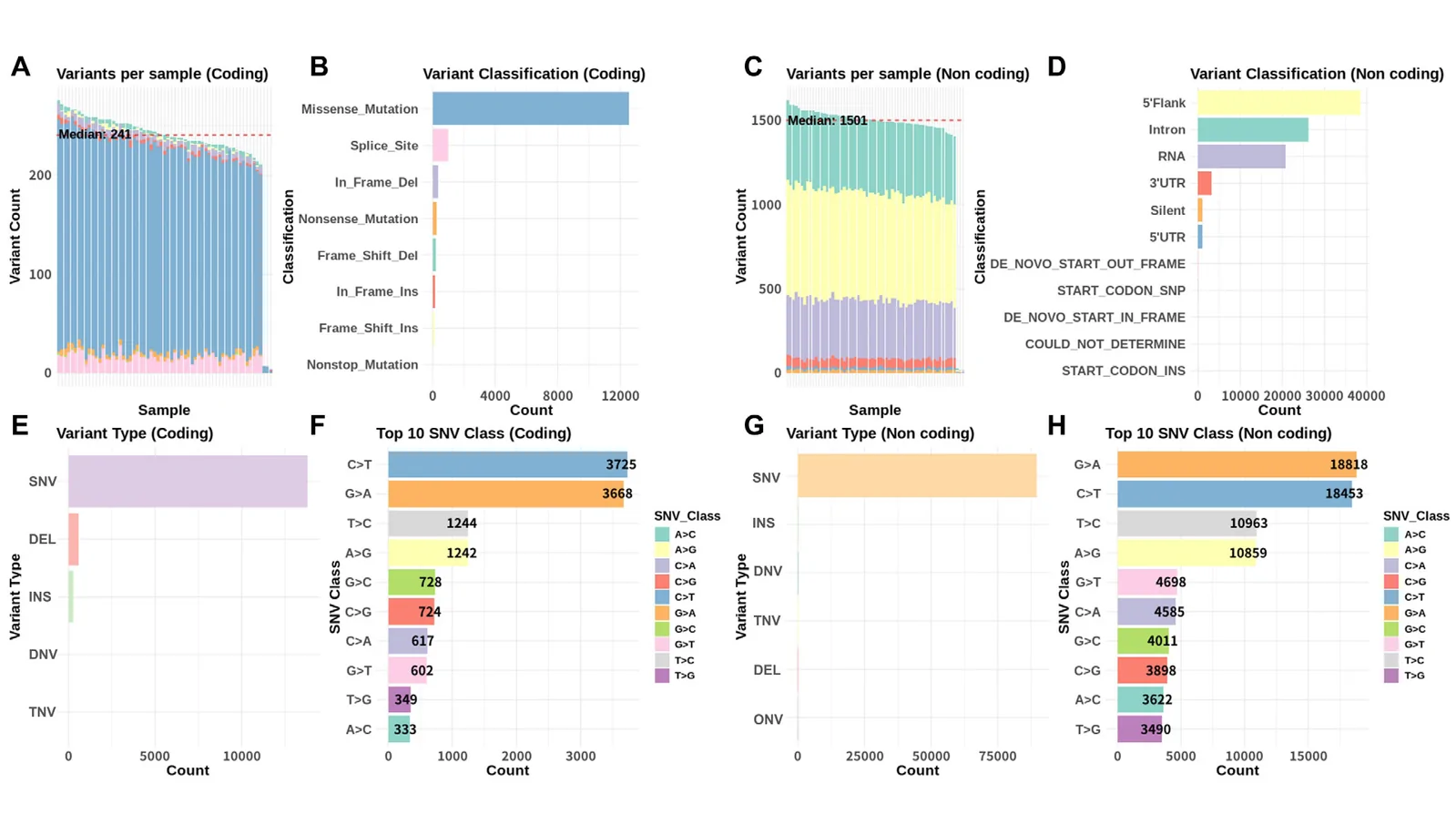
Distribution and classification of somatic variants in coding and non-coding regions. The color coding indicates different types and classes of variants. A: Sample-wise counts of coding-region variants; the median (241 variants) is indicated by a red dashed line. B: Proportional distribution of coding-region mutation classes: missense, splice-site, in-frame indel, nonsense, frameshift indel and nonstop. C: Sample-wise counts of non-coding-region variants; the median (1501 variants) is indicated by a red dashed line. D: Proportional distribution of non-coding variant functional categories: 5′ flank, intron, RNA, UTR, silent and specialized classes. E: Breakdown of coding-region variant types: SNV, DEL, INS, DNV and TNV. F: Top ten single-nucleotide substitution classes in coding regions. G: Breakdown of non-coding-region variant types: SNV, INS, DEL, DNV, TNV and oligonucleotide variant (ONV). H: Top ten single-nucleotide substitution classes in non-coding regions.

In non-coding regions, the median SV count per individual was 1501 (range: 19–1619), with intronic and flanking regions accounting for the majority of variants (figure 2C,D). As in coding regions, SNV were the dominant variant type, and the most frequent substitution patterns were G>A and C>T (figure 2G,H). Overall, the cohort exhibited a median of 1744 SV per worker, with detailed distributions summarized in supplementary table S2.

To explore underlying mutational processes, mutational signature analysis was performed using non-negative matrix factorization based on 96 trinucleotide mutation contexts. Participants were stratified into low- and high-dose groups according to the median cumulative radiation dose (25.31 mSv). The extracted signature from the low-dose group showed the greatest similarity to COSMIC SBS5 (cosine similarity=0.789), whereas the extracted signature from the high-dose group showed the greatest similarity to COSMIC SBS6 (cosine similarity=0.769) (supplementary figure S1). To facilitate evaluation of the mutational signature analysis, the per-sample 96 trinucleotide mutation-context matrix and inferred COSMIC SBS contribution matrix are provided in Dataset S3. SBS5 is a clock-like signature of unknown etiology, while SBS6 is associated with defective DNA mismatch repair. However, given the relatively small blood-based cohort and the descriptive nature of cosine similarity matching, these findings should not be interpreted as definitive evidence of radiation-specific mutational processes. Rather, they suggest that different cumulative dose groups may show distinct mutational signature patterns.

### Correlation between cumulative radiation dose and somatic variant accumulation, and the influence of clinical factors

We next examined the relationship between cumulative radiation dose and SV accumulation. A statistically significant positive correlation was observed between cumulative dose and SV burden in coding regions (R=0.34, P=0.0077), non-coding regions (R=0.31, P=0.015), and overall SV counts (R=0.32, P=0.013) (figure 3A–C). In contrast, age was not significantly correlated with total SV burden (R=−0.073, P=0.57; figure 3D).

**Figure 3:**
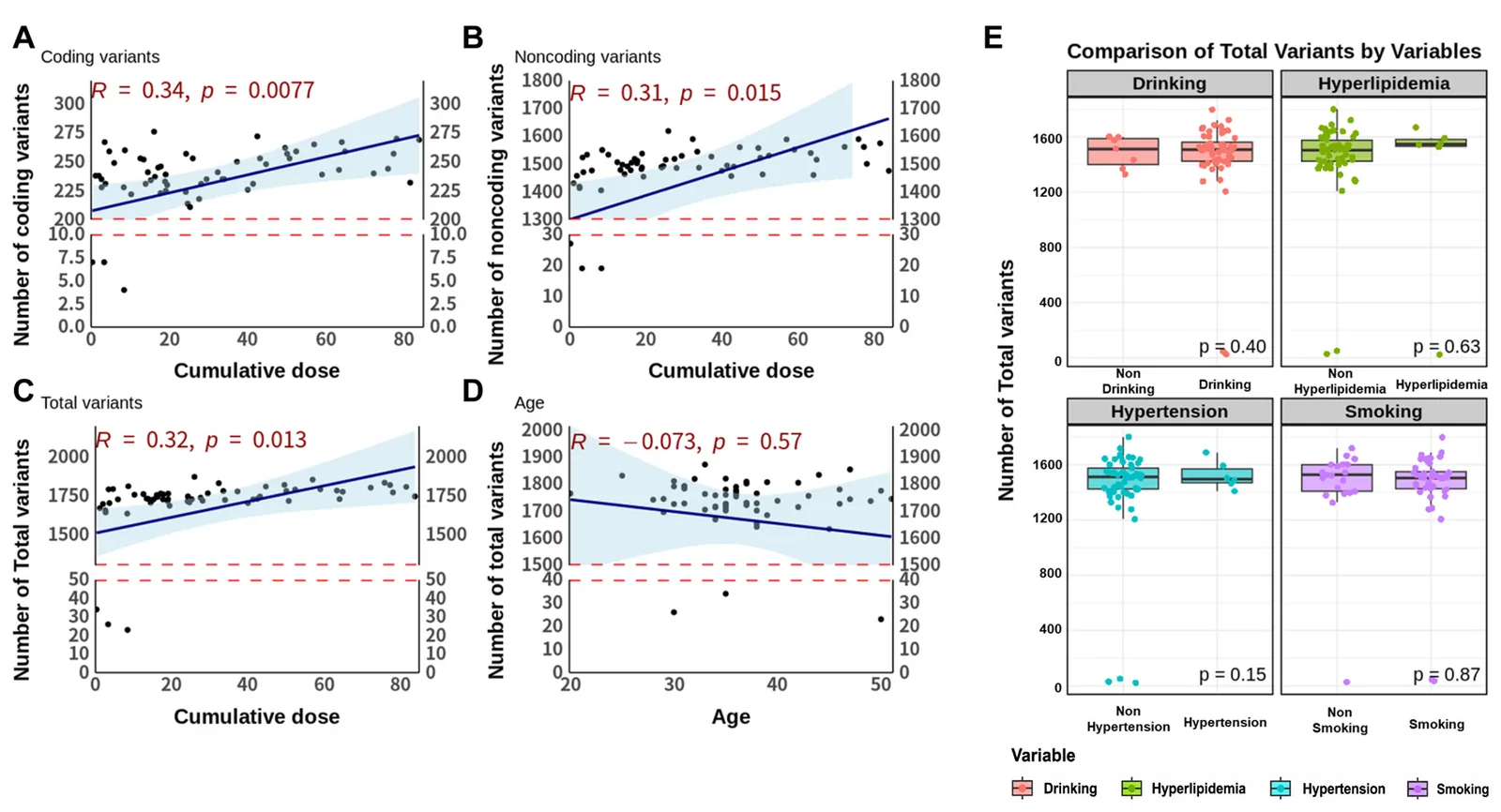
Correlation of cumulative radiation dose and clinical factors with somatic variants. A: Shows a positive correlation between the cumulative radiation dose and the number of coding variants (R=0.34, P=0.0077). The y-axis represents the number of coding variants, and the x-axis represents the cumulative radiation dose. B: Displays a positive correlation between the cumulative radiation dose and the number of non-coding variants (R=0.31,P=0.015). The y-axis represents the number of non-coding variants, and the x-axis represents the cumulative radiation dose. C: Indicates a positive correlation between the cumulative radiation dose and the total number of variants (R=0.32, P=0.013). The y-axis represents the total number of variants, and the x-axis represents the cumulative radiation dose. D: Shows no significant correlation between age and the total number of variants (R= -0.073, P=0.57). The y-axis represents the total number of variants, and the x-axis represents age. E: Compares the number of total variants across different clinical variables. The top left box plot compares non-drinkers and drinkers (P=0.40), the top right compares individuals with and without hyperlipidemia (P=0.63), the bottom left compares individuals with and without hypertension (P=0.15), and the bottom right compares non-smokers and smokers (P=0.87). A two-sided P< 0.05 threshold was used to define statistical significance.

Sensitivity analyses excluding three individuals with extremely low SV counts yielded consistent results, with significant positive correlations between cumulative dose and SV burden across coding, non-coding, and total variants (supplementary figure S2A–C). Age remained non-significant in these analyses (supplementary figure S2D).

Comparisons of SV burden across clinical subgroups showed no significant differences by smoking status, alcohol consumption, hypertension, or hyperlipidemia (figure 3E). In multivariable linear regression analysis adjusting for these covariates, none of the clinical factors demonstrated a statistically significant association with total SV count (table 2). The overall model explained a limited proportion of variance (R^2^=0.029), indicating that the evaluated clinical factors did not account for a substantial proportion of inter-individual variability in SV burden. However, because information on non-occupational radiation exposure and other potential health-related confounders was not comprehensively available, residual confounding cannot be excluded. Collectively, these results indicate a weak but consistent increase in somatic variant burden associated with cumulative low-dose ionizing radiation exposure in Korean radiation workers.

**Table 2:** Multiple linear regression analysis of clinical factors on the accumulation of somatic variants. [SE=standard error]

Variables	Reference group	Estimate	SE	T-value	P-value
Age ^a^ (years)	-	-0.5116	1.0137	-0.505	0.616
Smoking ^b^	Never	-0.3802	12.0673	-0.032	0.975
Drinking ^c^	Non-drinker	-7.2543	17.6987	-0.410	0.683
Hypertension ^d^	No	16.2099	19.5662	0.828	0.411
Hyperlipidemia ^d^	No	-13.1577	20.5415	-0.641	0.524

^a^ Age was treated as a continuous variable. ^b^ Smoking was categorized as binary (0=never smoker, 1=ever smoker). Individuals who currently smoke or have a history of smoking were classified as ‘smokers’. ^c^ Drinking was treated as a binary variable (0=non-drinker, 1=drinker). Participants who reported any alcohol consumption, including less than once per month, were considered drinkers. ^d^ Hypertension and hyperlipidemia were defined based on self-reported diagnosis or treatment history and treated as binary variables (0=no, 1=yes). The outcome variable was the total number of somatic mutations identified per individual.

## Discussion

This study investigated genomic alterations in 65 industrial radiographers (mean age: 36.7 years) with an average cumulative workplace radiation dose of 33.9 mSv and employment duration of 12.7 years. The most common type of genetic mutation observed was SNV, with the majority falling into the base substitution category, specifically C>T and G>A mutations. Base excision repair may be relevant when interpreting these base-substitution patterns, as it repairs small base lesions caused by oxidative and other DNA-damaging processes (26). Known disease-related or cancer-driver mutations were not identified based on computational annotation of coding variants; however, this should not be interpreted as a functional safety assessment, because no experimental functional validation was performed. Our results align with findings from WGS-based animal model studies, such as the work by Matsuda et al (7), which demonstrated dose-dependent increases in somatic SNV in mouse hematopoietic stem cells following X-ray exposure. Experimental evidence from genotoxic stress models also supports the importance of DNA damage repair pathways in maintaining genome stability (27). These results support the biological plausibility of radiation-associated genomic alterations even at relatively low occupational doses, while not establishing causality in the present cohort. Although the relatively small sample size, lack of a matched unexposed WGS control group, exclusive reliance on peripheral blood, and single-sample variant-calling framework limit causal attribution of the observed somatic variant burden and mutational signature patterns to occupational LDIR, our findings suggest that WGS-based somatic variant profiling may provide an exploratory framework for evaluating genomic alterations in occupationally exposed populations. In future longitudinal studies, somatic mutation burden and mu tational signature patterns may complement conventional physical dosimetry and cytogenetic biodosimetry, particularly for exploring radiation-sensitive genomic changes that may precede clinically detectable outcomes.

We observed a statistically significant but weak positive correlation between cumulative radiation dose and somatic variant burden, indicating a possible dose-related trend. This mutation burden could not be fully explained by common clinical variables such as age, smoking, alcohol consumption, hypertension, and hyperlipidemia. While our analysis did not reveal statistically significant associations between SV burden and common clinical variables such as age, smoking status, or alcohol consumption, these lifestyle-related factors may still contribute to genomic alterations and residual confounding cannot be excluded. Previous WGS-based studies have consistently demonstrated age-related increases in somatic mutational burden across various human tissues, including the brain, lung, skeletal muscle, and adipose tissue (28–31). In addition, smoking has been shown to significantly increase mutation frequencies in airway epithelial cells (29, 32). These findings underscore the need for cautious interpretation of our results and suggest that subtle mutagenic effects of aging and lifestyle factors may not have been fully captured in our cohort.

Although the mean cumulative dose in our cohort (33.9 mSv) is relatively low compared to ICRP’s occupational dose limits, growing evidence suggests that even low-dose ionizing radiation (LDIR) may elicit genomic alterations. In vitro experiments have demonstrated that somatic mutations can be induced at low doses of X-rays, with Nagashima et al (33) reporting dose-dependent increases in hypoxanthine-guanine phosphoribosyltransferase (HPRT) locus mutations following exposure above 0.15 Gy. Interestingly, the authors also noted that radiation doses below 0.1 Gy may not directly trigger DSB-related events, but could instead enhance spontaneous mutagenesis.

Supporting evidence from human cytogenetic studies indicates that low-level occupational radiation exposure may be associated with measurable chromosomal aberrations. Farkas et al (34) observed significantly higher frequencies of chromosomal aberrations and aberrant cells in nuclear medicine workers with a five-year cumulative dose of just 3.54 ± 4.95 mSv, when compared to those working in computed tomography (CT) departments. Likewise, Cherednichenko et al (35) reported a statistically significant increase in chromosomal aberration frequency among individuals chronically exposed to less than 10 mSv/year. These findings provide cytogenetic context for the possibility of measurable genomic effects in some low-dose occupationally exposed populations. However, the present WGS-based findings cannot establish that the observed somatic variants were induced by occupational radiation exposure.

An important point to note is that the mutational mechanism suggests a dose-response relationship without a discernible threshold. This concept is consistent with the stochastic model of radiation-induced cancer, which has been supported by epidemiological evidence (1, 3). Interestingly, radiation-induced point mutations tend to occur randomly throughout a gene, while spontaneous mutations tend to cluster at specific sites (36, 37). Our findings indicate a possible dose-dependent trend in somatic mutation burden, although the strength of the association is limited. This observation is generally consistent with previous reports suggesting a relationship between cumulative radiation exposure and increased mutational burden. Although previous studies have reported that radiation-induced point mutations may occur across various genomic regions, our findings should be interpreted cautiously because potential non-radiation occupational exposures were not comprehensively assessed in the present study.

Our findings are consistent with WGS-based studies in irradiated murine models. A significant increase in somatic SNV in hematopoietic stem cells following low-dose X-ray exposure has been reported (7). Dose-related changes in induced mutations have also been reported in offspring derived from irradiated mouse germ cells (5). Furthermore, a significant elevation in the frequency and an altered spectrum of de novo germline mutations in mouse offspring following parental exposure to ionizing radiation has likewise been documented (4).

Human studies of radiation-associated mutational signatures have mainly focused on radiation-associated malignancies or cancer patients exposed to radiotherapy (8, 9). By contrast, studies of exposed individuals without overt cancer have examined chromosome aberrations in peripheral blood lymphocytes (10), germline mutations in nuclear test veterans (11), and de novo mutations in offspring after paternal radiation exposure (12), rather than blood-derived somatic mutational signatures. Our study builds on this evidence by focusing specifically on healthy occupationally exposed individuals and applying stringent variant filtering methods.

The average somatic SNV burden in our cohort was approximately 1742 variants per person. Previous WGS-based studies of normal human tissues have reported variable somatic mutation burdens across tissue types, cell types, ages, sequencing strategies, and variant-calling approaches, with estimates ranging from hundreds to thousands of somatic SNV per genome in adult tissues (28–31). Because these studies analyzed different tissues, including brain, bronchial epithelium, peripheral blood-derived cells, and skeletal muscle satellite cells, direct comparison with the present blood-based cohort should be interpreted cautiously.

Recent epidemiological studies have reported associations between low-dose or protracted ionizing radiation exposure and long-term health outcomes, particularly cancer, although evidence varies by outcome and exposure range (15, 37–40). These studies provide important public health context for occupational LDIR research but do not directly address whether cumulative low-dose exposure is associated with blood-derived somatic variant burden. Therefore, our WGS-based findings should be interpreted as exploratory biological evidence that requires validation in larger longitudinal cohorts with comprehensive exposure and health-outcome data.

Technical artifacts related to WGS, including sequencing errors, mapping inaccuracies, and batch effects, may have influenced variant calling despite the use of standard quality filters and manual review. Although these procedures were applied to reduce false-positive calls, residual technical bias cannot be completely excluded and should be considered when interpreting the observed somatic variant burden.

This study has several limitations. First, the sample size was small and all participants were Korean industrial radiographers, which limits statistical power and generalizability. Second, the absence of matched unexposed controls limits causal attribution of the observed somatic variant burden to occupational radiation exposure. Third, because the study relied on peripheral blood samples and a single-sample analytical framework, residual germline contamination and hematopoietic clonal processes cannot be completely excluded. Fourth, p rotential confounding factors, including non-occupational radiation exposure, medication histories, underlying inflammatory conditions, lifestyle-related factors, and other occupational exposures, were not comprehensively captured. Therefore, the observed association between cumulative occupational dose and somatic variant burden should be interpreted as exploratory rather than causal. Larger longitudinal studies with matched controls, paired germline reference samples, and comprehensive exposure assessment are needed to validate these findings.

In conclusion, this WGS-based study of Korean industrial radiographers observed a weak positive association between cumulative occupational radiation dose and blood-derived somatic variant burden, while the overall mutation burden remained within the expected range for healthy individuals. Given the small sample size, absence of matched unexposed controls, and blood-only study design, these findings should be interpreted as hypothesis-generating rather than definitive evidence of radiation-induced genomic damage. Larger longitudinal studies incorporating exposed and unexposed populations, paired germline reference samples, and comprehensive occupational and radiation exposure assessment are needed to clarify the relationship between chronic low-dose occupational radiation exposure and somatic mutational processes.

## Supplementary material

Supplementary material 1 Supplementary material

## Data Availability

The data supporting the findings of this study are available in the supplementary materials (www.sjweh.fi/article/4317) and the Sequence Read Archive (SRA) database (accession number: PRJNA1153334).

## Code availability

All computational codes used in this study are publicly available at the following GitHub repository: https://github.com/kangjunho/Genomic-Insights-into-Somatic-Mutations-from-Occupational-Exposure-to-Low-Dose-Ionizing-Radiation
