# Supplementary material for: Genomic insights into somatic mutations from occupational exposure to low-dose ionizing radiation
Source: Scand J Work Environ Health. 2026 Oct 26;52(6):661–71. doi: 10.5271/sjweh.4317 (PMC13732785; doi:10.5271/sjweh.4317)
Supplement: Supplementary file 1 [file SJWEH-52-6-661-s001.pdf]

Genomic insights into somatic mutations from occupational exposure to low-dose ionizing radiation[1]

by Kihun Kim, MD, PhD, Junho Kang, PhD, Dong Min Lim, PhD, Seongjae Jang, PhD, Younghyun Lee, PhD, Yang Hee Lee, BS, Hyo Jin Yoon, BS, SuSan Yang, MS, Seung Hyun Kim, BS, Dalnim Lee, PhD, Won Il Jang, MD, PhD, Hyoju Sung, PhD, Min-Jung Kim, PhD, Eunjeong Son, MD,10 Hyung-Sik Kim, PhD, Dongjun Lee, PhD, Hae Ryoun Park, DDS, PhD, Songwon Seo, PhD,[2] Yun Hak Kim, MD, PhD [2]

1. Supplementary material
2. Correspondence to: Dr. Songwon Seo, National Radiation Emergency Medical Center, Korea Institute of Radiological and Medical Sciences, 75 Nowon gil, Nowon-gu, Seoul, 01812, Republic of Korea. [E-mail: seo@kirams.re.kr]; Prof. Yun Hak Kim, Department of Anatomy and Department of Biomedical Informatics, School of Medicine, Pusan National University, 49 Busandaehak-ro, Yangsan 50612, Republic of Korea. [E-mail: yunhak10510@pusan.ac.kr]

Table S1. Radiation exposure metrics for study participants (n=65)

Variables	Mean (mSv)	SD (mSv)
**Cumulative dose (year)**		
0.5	0.609	1.790
1	1.252	3.335
3	3.952	6.286
5	11.346	15.727
**Dose_mean_DC**	0.070	0.040
**Dose_min_DC**	0.006	0.010
**Dose_max_DC**	0.265	0.175
**Dose_mean_FISH**	0.234	0.141
**Dose_min_FISH**	0.256	0.425
**Dose_max_FISH**	0.832	0.282

*DC, dicentric chromosome assay; FISH, fluorescence in situ hybridization translocation assay; SD, standard deviation.

Radiation exposure was characterized by integrating physical dosimetry with cytogenetic biodosimetry. External doses measured by personal dosimeters were aggregated as cumulative exposures over four predefined intervals (0.5, 1, 3, and 5 years). Concurrently, biological dose estimates were obtained from peripheral blood lymphocyte assays: dicentric chromosome analysis yielded mean (Dose_mean_DC), minimum (Dose_min_DC), and maximum (Dose_max_DC) absorbed-dose estimates, whereas fluorescence in situ hybridization (FISH) translocation assays provided corresponding metrics (Dose_mean_FISH, Dose_min_FISH, Dose_max_FISH) (13).

Table S2. Distribution of somatic variants in Korean radiation workers.

Variant classification	Variant type	Number of variants
**Coding regions**		14,646
Missense	SNV	12,578
Splice site	SNV	985
Inframe deletion	DEL	363
Nonsense	SNV	242
Frameshift deletion	DEL	209
Inframe insertion	INS	157
Frameshift insertion	INS	95
Nonstop	SNV	17
**Noncoding regions**		90,524
5` Flank	Multiple	38,497
Intron	SNV	26,092
RNA	SNV	20,818
3` UTR	SNV	3,130
Silent	SNV	1,028
5` UTR	SNV	929
De nove start out-of-frame	DEL	15
Start codon	SNV	8
De novo start inframe	INS	4
Could not determine	-	2
Start codon insertion	INS	1

*SNV: single nucleotide variant, INS: insertion, DEL: deletion

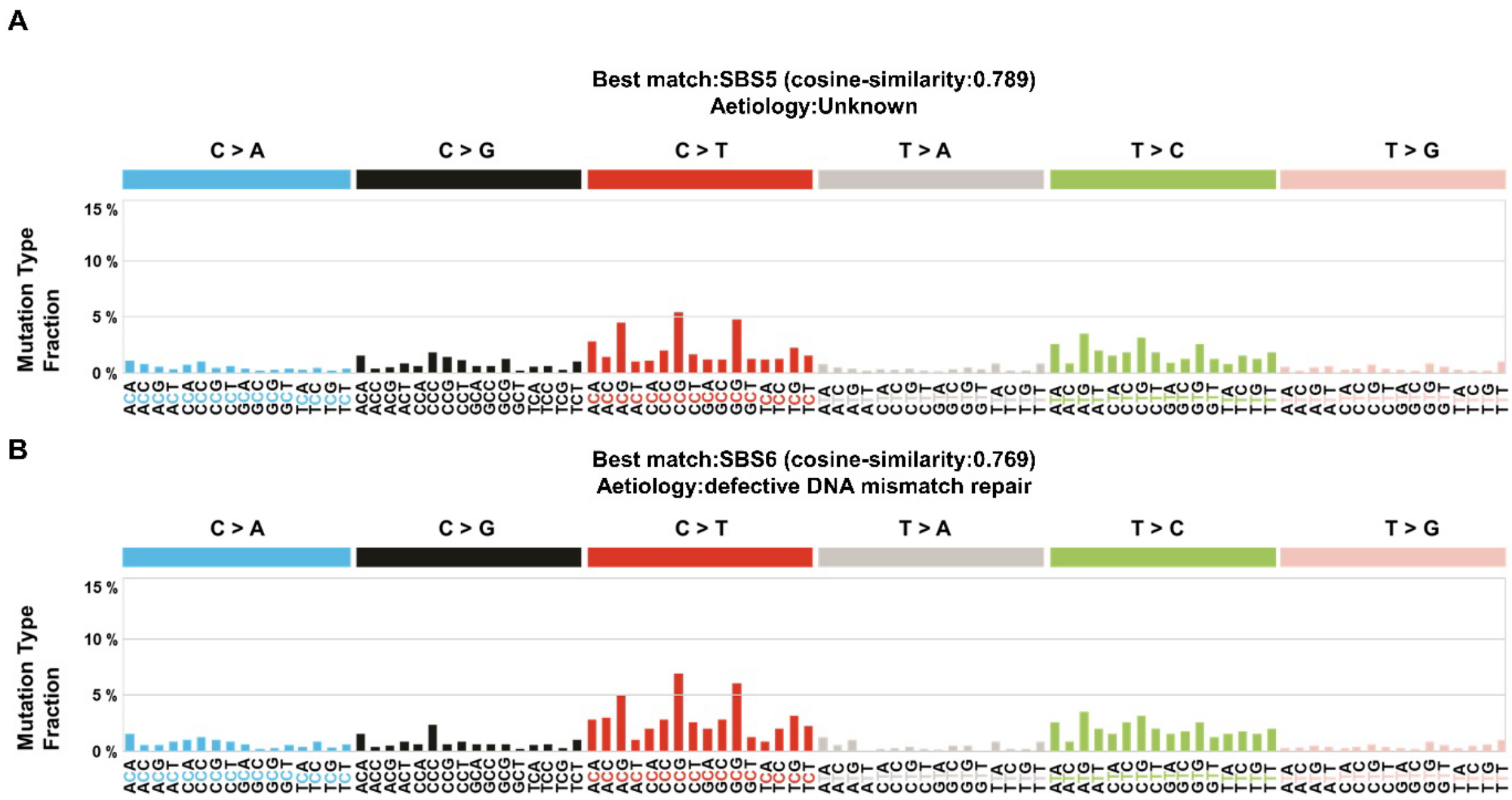

Fig. S1. Mutational signature analysis of somatic variants in Korean radiation workers. Mutational signatures were extracted using non-negative matrix factorization based on the trinucleotide context of single-nucleotide variants. (A) Mutational profile of the low-dose group across 96 trinucleotide contexts. The extracted signature showed the highest cosine similarity to COSMIC SBS5 (cosine similarity = 0.789), a clock-like signature of unknown aetiology. (B) Mutational profile of the high-dose group across 96 trinucleotide contexts. The extracted signature showed the highest cosine similarity to COSMIC SBS6 (cosine similarity = 0.769), which is associated with defective DNA mismatch repair.

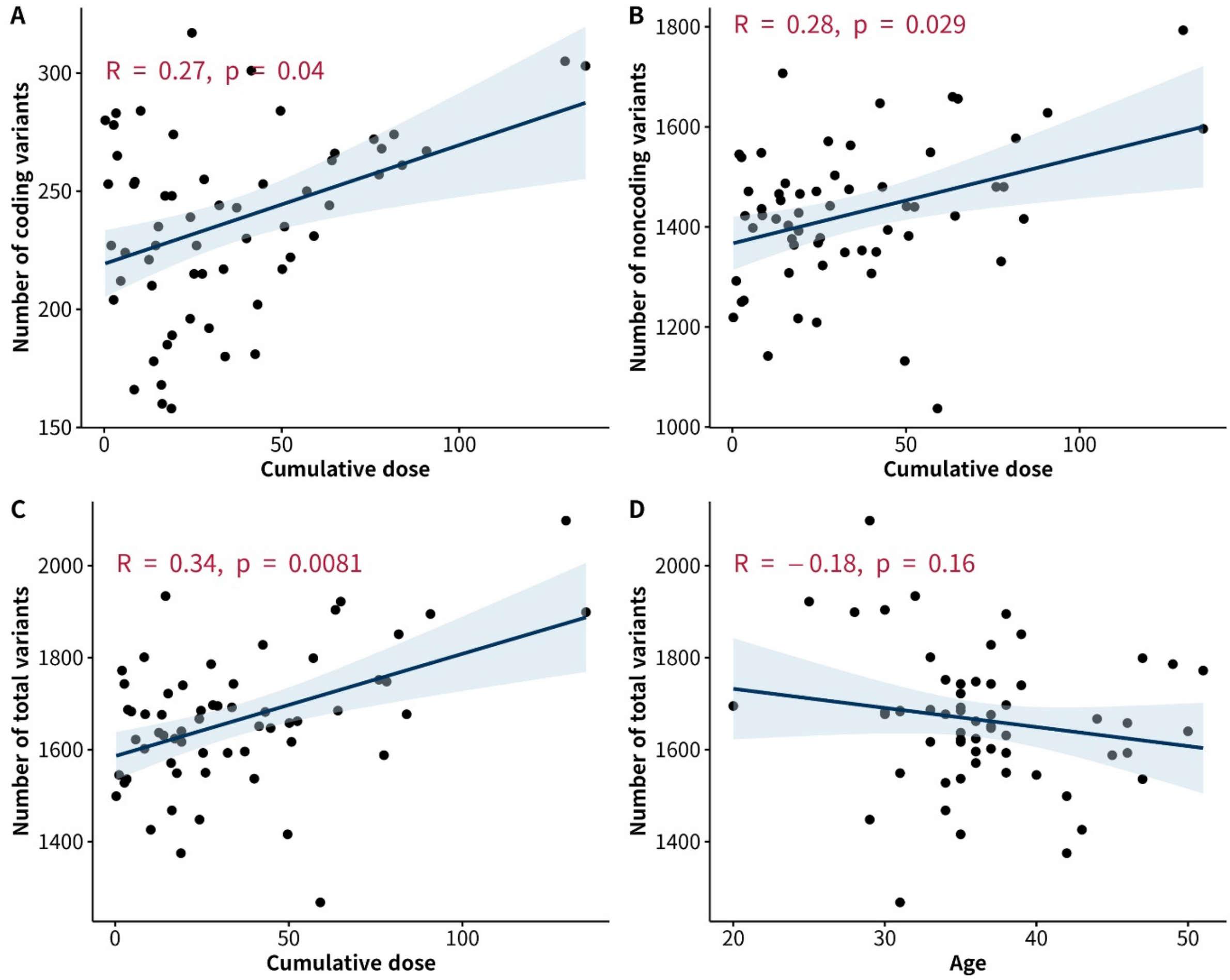

Fig. S2 Sensitivity Analysis of the Correlation Between Cumulative Radiation Dose and Somatic Variants After Outlier Removal.

The figure presents the results of a sensitivity analysis excluding three outlier data points (total SVs < 50) to assess the robustness of the association between cumulative radiation dose and somatic variant burden. A: Displays the correlation between cumulative radiation dose and the number of coding variants after outlier removal ($R = 0.27$, $p = 0.04$). The y-axis represents the number of coding variants, and the x-axis represents the cumulative radiation dose. B: Shows the correlation between cumulative radiation dose and the number of non-coding variants ($R = 0.28$, $p = 0.029$). C: Indicates a positive correlation between cumulative radiation dose and the total number of somatic variants ($R = 0.34$, $p = 0.0081$). D: Illustrates no significant correlation

between age and the total number of variants ($R = -0.18$, $p = 0.16$). A two-sided $p < 0.05$ threshold was used to define statistical significance.
